# Do African American adolescents internalize direct online discrimination? Moderating effects of vicarious online discrimination, parental technological attitudes, and racial identity centrality

**DOI:** 10.3389/fpsyg.2022.862557

**Published:** 2022-09-13

**Authors:** Chun Tao, Kimberly A. Scott

**Affiliations:** ^1^Department of Psychiatry and Psychology, Mayo Clinic, Scottsdale, AZ, United States; ^2^Counseling and Counseling Psychology, Arizona State University, Tempe, AZ, United States; ^3^School of Social Transformation, The Center for Gender Equity in Science and Technology, Arizona State University, Tempe, AZ, United States

**Keywords:** African American adolescents, direct online discrimination, internalized computing stereotypes, parental technological attitudes, racial identity centrality

## Abstract

African American adolescents have become more active users of digital media, which may increasingly expose them to direct online discrimination based on their racial and gender identities. Despite well-documented impacts of offline discrimination, our understanding of if and how direct online discrimination affects African American adolescents similarly remains limited. Guided by intersectional and ecological frameworks, we examined the association between direct online discrimination and internalized computing stereotypes in African American adolescents. Further, we explored the moderating effects of systemic and individual factors – vicarious online discrimination, parental technological attitudes, and racial identity centrality – on this association by adolescent gender. Utilizing data from 1041 African American parent-adolescent dyads, we found a positive association between adolescents’ direct online discrimination and internalized computing stereotypes. Surprisingly, greater vicarious online discrimination mitigated this association for both male and female adolescents. Further, parental technological attitudes and racial identity centrality mitigated this association only for female but not male adolescents. Our findings highlight the importance of understanding the impact of media on adolescents’ online experiences from intersectional and systemic perspectives. We discuss the implications for prospective research and educational programs focused on African American adolescents’ digital media use and online experiences.

## Introduction

It is not uncommon for African American adolescents to experience discrimination based on their racial and gender identities in society. As African American adolescents are becoming more active users of digital and social media (e.g., Rideout et al., 2011; Tynes and Mitchell, 2014; Nielsen, 2016), they, on one hand, are enhancing their confidence and abilities in technological innovation, and on the other hand, may be increasingly exposed to discrimination based on their identities in an online setting, namely online discrimination (Bliuc et al., 2018; Keum and Miller, 2018). Online communication is unique compared to face-to-face or offline encounters such that adolescents may be more willing to disclose their identities (e.g., racial and gender background) whereas individuals who perpetuate discrimination may censor less on their expressions given greater anonymity (Joinson, 2001; Gray, 2012; Keum and Miller, 2018), which poise adolescents for higher risk of being mistreated (Kahn et al., 2013).

Offline discrimination perceived by African American adolescents has been well-documented to link to negative outcomes such as greater symptoms of depression and anxiety (e.g., Lambert et al., 2009; Priest et al., 2013) and lower self-esteem (e.g., Seaton, 2010). Similarly, adolescents may be vulnerable to the impact of online discrimination (Umaña-Taylor et al., 2015). They may adopt greater internalized computing stereotypes, defined as adolescents’ beliefs that confirm to negative views on the abilities of African Americans in general, and male and female African Americans specifically, to engage in technology or related jobs (Cheryan et al., 2013; Masters et al., 2021). Informed by an intersectional perspective (e.g., Crenshaw, 1991; McCall, 2001; Cho et al., 2013; Collins and Bilge, 2016), adolescents’ lived experiences online are uniquely shaped by multiple factors, such as their race, gender, and socioeconomic status. To this end, we conceptualized African American adolescents’ direct online discrimination as discrimination based on adolescents’ race and gender at the individual level given their prominence as social identities (Collins, 2015; Ireland et al., 2018). As such, the first goal of this study was to examine the association between African American adolescents’ direct online discrimination and internalized computing stereotypes. We also explored if this relation varied between male and female adolescents. In recognizing the political discourses around the term African American being, at times, different than Black American, we choose to use “African American” throughout this paper for consistency.

With continued advances in technology, the context in which African American adolescents grow and mature has been rapidly transforming, with media and cultural trends playing a more salient role (Steinke, 2017). Of significance is investigating systemic and individual factors that influence the internalization of direct online discrimination (Keum and Miller, 2018). Guided by the Ecological Systems Model (Bronfenbrenner and Morris, 1998) via an intersectional lens, the second goal of this study was to examine how factors at exosytem, microsystem, and individual levels may affect adolescents’ experiences with online discrimination. At the exosystem level, we predicted a magnifying effect of adolescents’ vicarious online discrimination, as defined by vicarious perceptions of discriminative content online that were not directly targeting adolescents personally (Tynes et al., 2010). At the microsystem level, we expected a mitigating effect of parental technological attitudes, or parents’ positive attitudes toward their adolescent children’s use of technology, as a moderating factor given important socialization messages parents would provide for adolescents’ development (McHale et al., 2006). At the individual level, we examined if adolescents’ racial identity centrality, defined as how important racial identity is to adolescents’ self-image, played an identical mitigating role for online racial discrimination (e.g., Umaña-Taylor et al., 2015; Moses et al., 2019). We also explored if these moderated associations varied by adolescent gender.

### Guiding frameworks

Originally coined by Kimberle Crenshaw (1989), intersectionality is useful as both a theoretical framework and methodological paradigm (e.g., Cole, 2009; Stevens-Watkins et al., 2014; Collins, 2015; Moradi and Grzanka, 2017; Ireland et al., 2018). As a theoretical lens, an intersectional approach requires more than a single unit of conceptualization to understand the interlocking dimensions of oppression (Moradi and Grzanka, 2017). Race alone cannot explain the totality of individuals’ experiences, particularly for groups historically marginalized such as African Americans. The contextualized meanings of intersecting sociocultural features, such as race, gender, and socioeconomic status, must be recognized as mutually constitutive, which grant individuals differential access to power, privilege, and oppression in society. African American women and female adolescents may be exposed to unique discriminatory portrayals of them as being oversexualized and promiscuous (e.g., Jezebel), self-sacrificing, and nurturing (West, 1995; Smith, 2005; Szymanski and Lewis, 2016), whereas African American men and male adolescents may be viewed as physically and sexually violent, gifted at sports, and uninvolved in their household (e.g., absent fathers; Schwing et al., 2013). In online spaces, African American male and female adolescents may differ not only in the content of their exposure to discrimination but also frequency. In fact, African American female adolescents have been found to receive more mistreatment based on their race and gender, especially sexualized victimization, than their male counterparts (e.g., Gray, 2012; Tynes and Mitchell, 2014).

Methodologically, intersectionality analyzes power dynamics in an inequitable system by studying how sociocultural identities are in relation to each other (Shields, 2008). It is critical to explore the individual, interpersonal, and structural relations which may ascribe to situated meanings (Collins and Bilge, 2016). That is, individual experiences for adolescents may interact with the oppression they face at the systemic level, which may further enact their agency to change and engage in actions to modify the internalization of online discrimination (Stevens-Watkins et al., 2014; Ireland et al., 2018). We drew upon these standpoints to examine how the online environment may manifest similarities and differences in African American male and female adolescents’ experiences at interpersonal and structural levels (Cole, 2009) through the Ecological Systems Model (Bronfenbrenner and Morris, 1998) with a broader intersectional lens.

The Ecological Systems Model (Bronfenbrenner and Morris, 1998) conceptualizes that adolescents do not grow in a vacuum but are instilled and shaped by the interactions among multiple systems with individuals. The layers of systems include the individual themselves, the microsystem (e.g., family, school, peers), the exosystem (e.g., mass media, local politics), the mesosystem (i.e., the interaction between the microsystem and the exosystem), and the macrosystem (e.g., attitudes and ideologies of the culture). In this study, we were interested in the systems that underlied the mass media (i.e., the exosystem) that contextualized online discrimination against African American adolescents. Specifically, we investigated the moderating effects of vicarious online discrimination (i.e., exosystem), parental technological attitudes (i.e., microsystem), and adolescents’ racial identity centrality (i.e., individual levels). We view the intersectional and ecological frameworks as interrelated as they afford us the opportunity to examine how contextual factors may affect adolescents’ online identities as being simultaneously raced and gendered and illustrate how the “matrix of domination” (Collins and Bilge, 2016) appears online.

### Direct online discrimination and internalized computing stereotypes

It is well-documented that perceived offline discrimination is associated with negative mental health outcomes such as greater symptoms of depression and anxiety in African American adolescents (e.g., Lambert et al., 2009; Priest et al., 2013). These experiences can also alter the way adolescents think about themselves in general (i.e., self-esteem; Thomas et al., 2004; Seaton, 2010), or more specifically manifested in internalized racism (e.g., thinking about their own race as inferior than others), sexism (e.g., thinking about their own gender as inferior), or both (e.g., thinking about their own race and gender as inferior; Clark et al., 1999).

Direct online discrimination has been posited to shed unique influences on well-being beyond the effects of offline discrimination (e.g., Umaña-Taylor et al., 2015; Tynes et al., 2016). The power dynamics online are unique compared to offline encounters, as researchers have pointed out that individuals may feel more ready to disclose radical and discriminatory viewpoints given a sense of anonymity, invisibility, and a lack of social and physical cues (e.g., discomfort by others in presence; Keum and Miller, 2018). Therefore, adolescents may be more vulnerable to direct and vicarious online discrimination, especially among those who spend more time online (Tynes et al., 2016, 2020). On the other hand, adolescents may have more opportunities to select and enter online spaces with people with similar backgrounds or beliefs (Keum and Miller, 2018). Due to these unique features and an increase in cyber-bullying and identity-based violence online in recent years, adolescents may intentionally and unintentionally perceive online discrimination in various forms more frequently (Smith, 2005; Barker and Jurasz, 2019; Wang et al., 2022). There has been emerging evidence on the negative effects of online discrimination on adolescents’ mental health outcomes (Tynes et al., 2016, 2020). However, our understanding of whether these impacts extend to adolescents’ confidence to traverse online platforms and their beliefs in the abilities of African Americans male and female African Americans to engage in technology (i.e., internalized computing stereotypes) remains limited.

Notably, the effects of online discrimination based on racial and gender identities may affect male and female adolescents differently. For example, Tynes et al. (2012) found that online racial discrimination was related to lower self-esteem only for female but not male adolescents. Despite that these findings were based on a relatively small sample of 125 African American adolescents, it is possible that due to the unique standpoints of marginalization for African American female adolescents, they may be more vulnerable to internalize discrimination than their male peers. This study aimed to analyze data from a larger sample to deepen our understanding of the implications of how race and gender may affect African American male and female adolescents’ internalization of direct online discrimination.

Drawing from prior findings on the negative implications of offline and online discrimination on self-esteem (Seaton, 2010; Tynes et al., 2012), we hypothesized that *there would be a positive association between direct online discrimination and internalized computing stereotypes such that the more frequently African American adolescents experience direct online discrimination, the greater they would report on internalized computing stereotypes (Hypothesis 1).* Due to limited existing evidence on gender differences, we left the question on whether this association was contingent upon adolescent gender exploratory. *Research Question 1: Does the association between direct online discrimination and internalized computing stereotypes differ between African American male and female adolescents?*

### Moderating effects of vicarious online discrimination on internalized computing stereotypes

As the Ecological Systems Model (Bronfenbrenner and Morris, 1998) highlights, the mass media and digital culture (i.e., exosystem) African American adolescents experience are critical to their development. On the Internet, over 71% of adolescents reported having personal or vicarious experiences of racial discrimination (Tynes et al., 2008) whereas more than 30% of adolescents reported experiences of cyberbullying (Ybarra and Mitchell, 2004). These statistics are not all-encompassing of the diverse ways discrimination can be perpetuated online and do not necessarily reflect what African American adolescents are experiencing nowadays. However, these findings imply that significantly more adolescents may be exposed to online discrimination vicariously than those who have personally experienced it.

Experimental research has found that if individuals, especially men who endorse higher hostile sexist beliefs, are exposed to sexist humor or music in a controlled environment, they are more likely perpetuate and tolerate sexism (Ford, 2000; Cobb and Boettcher, 2007). On the other hand, European American women who were exposed to a paradigm that suggested sexism was frequent rather than rare showed more threat responses and heightened anxiety post exposure, especially among those who more strongly identified with the group that is targeted by sexism (Eliezer et al., 2010). Together, these findings suggest having vicarious online discrimination to sexism may affect men and women’s response to future exposure to similar content; moreover, women may be more vulnerable to the negative physiological and emotional impacts of sexism if they were primed to believe sexism against their own group occurred frequently. These findings align with a priming paradigm such that previous exposures may poise individuals to memorize, perceive, and respond to identical content in a similar fashion in the future (Appiah, 2004; Dixon et al., 2009). It is possible that adolescents, irrespective of their gender, may become more susceptible to the impact of online discrimination against their race and gender if they perceive more frequent vicarious online discrimination because they identify with at least one of the identities (i.e., race, gender, or both) and become primed for future direct online discrimination.

Therefore, we hypothesized that *vicarious online discrimination would strengthen the hypothesized positive association between direct online discrimination and internalized computing stereotypes: compared to adolescents who reported less frequent vicarious online discrimination, those reporting more frequent vicarious online discrimination would report greater internalized computing stereotypes as they perceived more frequent direct online discrimination against themselves (Hypothesis 2a).* Findings on gender differences are equivocal, so we did not generate any hypothesis on the moderating effects of vicarious online discrimination by adolescent gender. *Research Question 2a: Does the interplay between vicarious online discrimination and direct online discrimination on internalized computing stereotypes vary depending on adolescent gender?*

### Moderating effects of parental technological attitudes on internalized computing stereotypes

At the microsystem level (Bronfenbrenner and Morris, 1998), parents can provide important socialization messages to adolescents’ development (McHale et al., 2006), shaping how adolescents develop a healthy self-identity, and view and respond to the realities of experiencing intersecting oppression, such as offline and online encounters of discrimination (i.e., racism and sexism; Edmondson et al., 1998; Richardson et al., 2015). This may occur through the process of gendered racial socialization for African American adolescents, through which parents provide messages to male and female adolescents based on their perceptions of the varied racial climate and landscape. Although scholars have highlighted the need to understand the unique socialization experiences of African American adolescents (West, 1995; Thomas and King, 2007), no known measure currently exists to assess gendered racial socialization for adolescents’ technological use. Parental technological attitudes may affect the socialization process as parents encourage or monitor African American male and female adolescents’ use of technology and online experiences. Kim and Davis (2017) found that from a diverse sample of adolescents’ perspectives, whether parents were more accepting vs. objecting (i.e., parental technological attitudes) and created rules on technological use were informed by parents’ knowledge and values in digital media. The more similar parents’ technological attitudes were to their early adolescent children’s attitudes, the more cohesive the parent-child relationships were, and the less likely adolescents behaved against rules their parents created (Kim and Davis, 2017).

Parental endorsement of positive technological attitudes (e.g., technology can present more opportunities) may not only promote parent-adolescent relationships but better prepare adolescents to face direct online discrimination. Adolescents whose parents view technology as more positive in general may view technology as more positive for adolescents’ development so that adolescents may have more opportunities to use technology and become competent, which may minimize the likelihood of them internalizing computing stereotypes. Parents who think positively about technology may also be more inclined to provide support as well as racial and gender socialization to assist adolescents to cope with potential vicarious and direct experiences of online discrimination, which in turn, are associated with greater academic confidence, performance, and self-esteem among African American adolescents (McHale et al., 2006; Murry et al., 2009; Alliman-Brissett and Turner, 2010).

Therefore, we expected that *parental technological attitudes would mitigate the hypothesized association between direct online discrimination and internalized computing stereotypes: for adolescents whose parents reported less positive technological attitudes, they would report greater internalized computing stereotypes as they perceived more frequent direct online discrimination whereas this association would be nonsignificant or negative for those whose parents with more positive attitudes (Hypothesis 2b).* Although researchers have attended to the variability in parenting values and behaviors between male and female adolescents in African American families (Smetana and Chuang, 2001; Skinner et al., 2016), the majority of existing research has focused on different parenting behaviors, such as more encouragement of autonomy toward adolescent sons and more strict monitoring of daughters (e.g., Varner and Mandara, 2013; Skinner et al., 2016; Tao et al., 2020). Significantly less attention has been paid to parental attitudes. Hence, we explored *would the interplay between parental technological attitudes and direct online discrimination on internalized computing stereotypes vary upon adolescent gender (Research Question 2b)?*

### Moderating effects of racial identity centrality on internalized computing stereotypes

At the individual level (Bronfenbrenner and Morris, 1998), adolescence has been considered as a transitional time for the development of racial and ethnic identity (Umaña-Taylor et al., 2014). From early to late adolescence, adolescents tend to reflect upon their lived experiences as they explore their racial background and develop a more complicated understanding of their racial identity (Umaña-Taylor et al., 2014). Racial identity has been known as a protective factor for perceptions of racial and gender discrimination in both face-to-face (e.g., Clark et al., 1999; Umaña-Taylor et al., 2015; Moses et al., 2019) and online contexts (Tynes et al., 2008). Racial identity centrality, one component of racial identity, reflects how important adolescents view their racial identity to themselves. African American adolescents’ beliefs and identity of self can be interwoven by portrayals of African Americans (or lack thereof) through media such as TV and the Internet (Allen, 1993, 2001; Dixon et al., 2009). Appiah (2004) found that African American college students’ ethnic identity affects how they traversed an online space such that those who endorsed a stronger ethnic identity spend more time reading and reviewing content targeting their racial and ethnic identity and prefer the content more than those who endorsed a weaker ethnic identity. Similar to their older counterparts in college, African American adolescents’ racial identity centrality may affect their online engagement and the potential internalization of direct online discrimination.

Based on how racial and ethnic identity has been found to influence African American adolescent’s online engagement and ability to effectively cope with online discrimination, we predicted that racial identity centrality may equip adolescents to maintain a healthy sense of self and community pertaining to technological abilities (i.e., lower internalized computing stereotypes). Specifically, *racial identity centrality would mitigate the hypothesized positive association between direct online discrimination and internalized computing stereotypes; that is, for adolescents who reported lower racial identity centrality, they would report greater internalized computing stereotypes if they perceived more frequent online discrimination against themselves whereas for those who reported higher racial identity centrality, this association would be nonsignificant or negative (Hypothesis 2c).* Moreover, we explored *would the interplay between racial identity centrality and direct online discrimination on internalized computing stereotypes differ depending on adolescent gender (Research Question 2c)?*

## Materials and methods

### Procedure

Data used in this study were collected as part of a national project that adopted a two-phase sequential exploratory design with two phases aimed to assess African American adolescents’ and their parents’ engagement in digital media from a dyadic approach. More details can be found in previous reports on different research questions (Rideout et al., 2016; Tao et al., 2020).

In the first phase, upon Institutional Review Board approval, we held ten semi-structured focus groups, six with African American parents and four with African American male and female adolescents, inquiring questions generated by a team of researchers led by the second author on adolescents’ digital media use. African American parents and adolescents were purposefully recruited for focus groups in five geographically and economically diverse regions – namely Oakland, California; Chicago, Illinois; New York City, New York; Atlanta, Georgia; and Shaw, Mississippi. The research team conducted thematic analyses of the focus group transcripts, which informed the development of survey items for the second phase.

In the second phase, families with at least one African American adolescent child between the ages of 11 and 17 years old across the United States were recruited using a combination of probability-based and nonprobability sampling (Rideout et al., 2016) in the fall of 2015. To enhance representation of the sample of adolescents in this study, we first utilized “KnowledgePanel, a probability-based web panel designed to be representative of the United States” (Rideout et al., 2016, p. 30) using addresses across the nation, followed by nonprobability or convenience sampling to collect data from a larger sample size. One parent and one eligible adolescent within each family participated in this study via phone contact. Parents provided consent for themselves and their adolescent child, and then answered questions about their demographic information, technological attitudes, and other measures beyond the scope of this study. Following parents’ responses, adolescents answered questions about their demographic information, direct and vicarious online discrimination, internalized computing stereotypes, and racial identity centrality. Parents and adolescents were compensated for their time of participation.

Throughout this project, an expert advisory group, including one researcher well versed in large-scale survey development, one psychometrician, two researchers experienced in research on African Americans, digital media, and culture, and one researcher with expertise in conducting digital media studies with Latinx Americans, was consulted. They provided feedback on the design and revision of interview questions in the first phase as well as the face validity and readability of survey questions in the second phase.

### Participants

A total of 1,041 African American parent-adolescent dyads (*n* = 2,082 individuals) participated in this study, including 341 dyads recruited from probability-based sampling and 700 dyads recruited from nonprobability sampling. Average age of adolescents was 14.06 years (SD = 1.98, range = 11–17) and average age of parents was 41.49 years (SD = 8.60, range = 30–72). Regarding gender, 522 male and 519 female adolescents, and 288 fathers and 753 mothers participated, resulting in 146 father-son dyads, 142 father-daughter dyads, 376 mother-son dyads, and 377 mother-daughter dyads. Participants resided in urban (45%), suburban (44%), and rural (11%) settings across the Northwest (18%), Midwest (17%), South (56%), and West (9%) regions in the United States. The sample included 34% parents who have reportedly had some high school education or high school diploma, 27% households with an annual income less than $25,000, and 41% single-parent households.

### Measures

#### Internalized computing stereotypes

Adolescents responded to four items that were created to evaluate their internalized computing stereotypes based on their racial and gender identities. These items pertained to the themes emerged from the first phase of the larger project as adolescents and parents discussed their beliefs about whether and how race and gender of African Americans or Black people play a role in their competence and success in computer-related tasks and careers compared to others of different racial, gender identities, or both. The research team used language to reflect the intersecting identities of adolescents based on race and gender (e.g., Black people, Black boys/girls, and Black women/men). Specifically, the four items administered were (1)“Black people are just as good at using computers as people of other races,” (2) “Black people are just as successful at computer-related jobs as people of other races,” (3) “Black girls are just as good as Black boys at using computers,” and (4) “Black women are just as successful as Black men at computer-related jobs.” Adolescents rated their agreement to each statement based on a four-point scale (1 = strongly disagree, 2 = disagree, 3 = agree, and 4 = strongly agree). All four items were reverse coded and mean scores across the four items were calculated with higher mean scores reflecting greater endorsements of computing stereotypes against Black people in general and Black women specifically. The four items demonstrated satisfactory internal reliability, Cronbach’s α = 0.88.

#### Direct online discrimination

Two items were created to assess the frequency of direct online discrimination: (1) “In the past year, how often have you personally been mistreated or disrespected online because of your race or ethnicity”; and (2) “In the past year, how often have you personally been mistreated or disrespected online because of your gender.” These items were generated based on themes emerged from the focus groups in the first phase on adolescents’ encounters of direct online discrimination based on their race or gender. Of note, a few adolescents shared in the focus groups that they at times were unsure if the discriminatory content they perceived targeted at their race, gender, or both. To be more inclusive of the assessment for both male and female adolescents, we chose to create two items, one for race and the other for gender, instead of one item based on their race and gender. In the second phase, adolescents rated these two statements on a four-point scale (1 = never, 2 = hardly ever, 3 = sometimes, and 4 = often). Higher scores reflected more frequent direct online discrimination. The two items were highly correlated, suggesting good reliability for a two-item measure, *r* = 0.82.

#### Vicarious online discrimination

Adolescents reported their vicarious online discrimination on four items developed in this project to assess their vicarious online discrimination to content that was disrespectful to groups based on Black people’s race, gender, and both. The items were created to reflect the interlocking identities as discussed in focus groups and in literature (e.g., Moradi and Grzanka, 2017). Participants used the same four-point scale as direct online discrimination to rate “How often if ever, do you see content online that is disrespectful to each of the following groups: (1) Black people in general; (2) women in general; (3) Black women in particular; and (4) Black men in particular.” Adolescents responded on a four-point scale (1 = never, 2 = hardly ever, 3 = sometimes, and 4 = often). Mean scores across the four items were computed with higher scores representing more frequent vicarious online discrimination. The four items showed excellent internal reliability, Cronbach’s α = 0.95.

#### Parental technological attitudes

Parents reported their attitudes toward technology on three items: (1) “The Internet exposes my child to important new ideas and information”; (2) “Computers offer my child new and interesting ways to express him/herself”; and (3) “Mobile devices such as smartphones and tablets offer my child new and interesting ways to express him/herself.” These items were created based on thematic findings from parental focus groups such that parents discussed that despite potential risk with negative content online, their adolescent children may benefit from using computers and the Internet for personal expressions and learning. In the second phase, parents indicated their agreements with each statement on a four-point scale (1 = strongly disagree, 2 = somewhat disagree, 3 = somewhat agree, and 4 = strongly agree). Mean scores were computed with higher scores indicating more positive parental technological attitudes. Internal reliability among the three items was good, Cronbach’s α = 0.82.

#### Racial identity centrality

Adolescents rated their agreement with one single-item statement – “In general, being an African American or Black person is an important part of my self-image” on a four-point scale (1 = strongly disagree, 2 = somewhat disagree, 3 = somewhat agree, and 4 = strongly agree). The single item was developed based on themes emerged from the first phase of this project on how important and central African American adolescents’ racial identities were to them. Higher scores on the item used in this study reflected higher racial identity centrality. Despite being a single-item measure, this item was identical to the items used to assess racial identity centrality on the Centrality Subscale of the Multidimensional Inventory of Black Identity for African American Adolescents (MIBI-T; Scottham et al., 2009). For reference, items on the Centrality Subscale of MIBI-T are “I feel close to other Black people,” “I have a strong sense of belonging to other Black people,” and “If I were to describe myself to someone, one of the first things that I would say is that I’m Black.” Items on this subscale assess if being a Black person is an important part of one’s self-image or self-identity, similar to the single-item measure on racial identity centrality used for brevity in this study.

### Data analytical strategies

We first conducted missing data analysis using Little’s MCAR Test. We then generated descriptive statistics and assessed data distribution of the main variables of interests in this study by adolescent gender. Next, we performed independent-samples *t*-tests to examine whether the main variables of interests vary in the scores by adolescent gender. Subsequently, to examine Hypothesis 1 and Research Question 1, we ran a hierarchical regression model with internalized computing stereotypes as the criterion. In Step 1 of the model, direct online discrimination and adolescent gender were entered; in Step 2, an interaction term between the two was entered. To further examine the rest of the hypotheses and research questions, we ran three independent regression models using the PROCESS macro, model 3, for SPSS (Hayes, 2013). In each model, internalized computing stereotypes were regressed on the independent variable (i.e., direct online discrimination), the respective moderator (i.e., vicarious online discrimination, parental technological attitudes, and adolescents’ racial identity centrality), adolescent gender, three two-way interaction terms (e.g., independent variable × moderator, independent variable × adolescent gender, moderator × adolescent gender), and one three-way interaction term (i.e., independent variable × moderator × adolescent gender). Effect sizes of the regression model were estimated by Cohen’s *f*^2^ and effect sizes of coefficients were estimated by standardized beta’s (*B*). To facilitate interpretations, the independent variable and all continuous moderators were mean centered and adolescent gender was effect coded (-1 = female, 1 = male). Significant interactions were decomposed with continuous variables being probed at mean, one standard deviation below and above the mean (Aiken and West, 1991).

## Results

### Preliminary analyses

All items assessing main variables of interest in this study had less than 2% of missing data except for the third item on the parental technological attitudes measure (3.6% missing). We performed a Little’s MCAR Test and results revealed that data were missing completely at random, χ^2^(78) = 94.45, *p* = 0.10. Notably, examinations on skewness and kurtosis demonstrated that responses on internalized computing stereotypes reflected a highly, negatively skewed and leptokurtic (peaked) distribution (skewness = 3.13 and kurtosis = 11.38), whereas responses on adolescents’ racial identity centrality reflected a highly, positively skewed and leptokurtic distribution (skewness = -2.63 and kurtosis = 7.17). A two-step transformation (Templeton, 2011) was performed to normalize the distribution of internalized computing stereotypes, resulting in decreases in skewness to 1.59 and kurtosis to 1.30. The transformed scores of internalized computing stereotypes (*M* = 1.25, SD = 0.36, range = 1.06 to 2.51) were highly correlated with the original scores, *r* = 0.93. We ran preliminary and regression analyses based on the original and transformed scores; because results are similar and that researchers have raised methodological concerns about the use of data transformation to adjust for data distribution (e.g., Feng et al., 2012), we reported findings based on original scores to allow meaning interpretations of regression coefficients.

Descriptive statistics and correlations of the main continuous variables of interest by adolescent gender can be found in Table 1. Independent-samples *t*-tests showed that African male adolescents (*M* = 1.25, SD = 0.56) reported greater internalized computing stereotypes than their female counterparts (*M* = 1.17, SD = 0.42), *t*(954) = 2.63, *p* = 0.009, although the effect size was small, Cohen’s *d* = 0.16. Male and female adolescents did not differ in their reports of direct online discrimination, vicarious online discrimination, or racial identity centrality. Parents of African American male and female adolescents did not differ in technological attitudes.

**TABLE 1:** Descriptive statistics and correlations of main continuous variables of interest by adolescent gender.

Variable	*r*					*M*(SD)			Range
			
	1.	2.	3.	4.	5.	Total	Male	Female	
1. Internalized computing stereotypes	–	0.12**	–0.05	–0.16***	–0.35***	1.21 (0.49)	1.25 (0.56)	1.17 (0.42)	1–4
2. Direct online discrimination	0.07	–	0.036***	0.12**	<0.001	1.67 (0.88)	1.66 (0.86)	1.68 (0.89)	1–4
3. Vicarious online discrimination	–0.12**	32***	–	0.18***	0.12**	2.89 (0.88)	2.86 (0.89)	2.91 (0.87)	1–4
4. Parental technological attitudes	–0.11*	–0.06	0.06	–	0.14**	3.46 (0.55)	3.46 (0.56)	3.46 (0.53)	1–4
5. Racial identity centrality	–0.64***	0.08	0.14**	0.05	–	3.74 (0.60)	3.72 (0.62)	3.75 (0.58)	1–4

Correlations for male adolescents are below the diagonal and correlations for female adolescents are above the diagonal.

*p < 0.05. **p < 0.01. ***p < 0.001.

### Direct online discrimination and internalized computing stereotypes

Hypothesis 1 predicted a positive association between direct online discrimination and internalized computing stereotypes. Regression analysis revealed that direct online discrimination explained 2% of variance in internalized computing stereotypes, *R*^2^ = 0.02, *F*(1, 1,023) = 18.32, *p* < 0.001, although the effect size was small, Cohen’s *f*^2^ = 0.01, power = 1.00. Adolescents who experienced direct online discrimination more frequently also endorsed higher internalized computing stereotypes, *b* = 0.05, *B* = 0.13, *t*(1,023) = 4.28, *p* < 0.001, which supported Hypothesis 1. We also explored if the association between direct online discrimination and internalized computing stereotypes would differ between African American male and female adolescents (Research Question 1). We found that the Step 2 of the regression model with adolescent gender added as a moderator did not explain significantly more variance in internalized computing stereotypes than Step 1, Δ*R*^2^ < 0.01, *F*(1, 1,021) = 0.08, *p* = 0.78, Cohen’s *f*^2^ < 0.01, power = 0.05. That is, the positive direct online discrimination-internalized computing stereotype relation held true for both male and female adolescents.

### Moderating effects

Results of the three moderation models to examine Hypotheses 2a–2c and Research Questions 2a–2c can be found in Table 2. Given multiple analyses were conducted simultaneously, significance level for meaningful interpretations was adjusted to 0.01.

**TABLE 2:** Regression coefficients for the effects of direct online discrimination by vicarious online discrimination, parental technological attitudes and racial identity centrality on adolescents’ transformed internalized computing stereotypes.

	Vicarious online discrimination		Parental technological attitudes		Racial identity centrality	
			
	*b*	SE	*b*	SE	*b*	SE
Intercept	1.21***	0.01	1.19***	0.01	1.18***	0.01
Direct online discrimination	0.09***	0.01	0.03**	0.01	0.03	0.01
Moderator (M)	–0.10***	0.01	–0.14***	0.01	–0.34***	0.02
Gender*^a^*	0.04**	0.01	0.03*	0.01	0.03*	0.01
Direct online discrimination × M	–0.08***	0.02	–0.05	0.01	–0.01	0.02
Direct online discrimination × Gender	0.01	0.01	–0.03	0.01	–0.01	0.01
M × Gender	–0.04*	0.01	–0.01	0.01	–0.09***	0.01
Direct online discrimination × M × Gender	–0.04	0.02	0.08**	0.01	0.11***	0.01
*R* ^2^	0.05***		0.04***		0.25***	
*N*	1,026		1,027		1,026	

^a^Gender here denotes adolescent gender and was effect coded as male = 1 and female = −1.

*p < 0.05. **p < 0.01. ***p < 0.001.

#### Moderating effects of vicarious online discrimination

Hypothesis 2a predicted that vicarious online discrimination would strengthen the positive association between direct online discrimination and internalized computing stereotypes. The regression model with vicarious online discrimination entered as one of the moderators explained 5% of variance in internalized computing stereotypes, *R*^2^ = 0.05, *F*(7, 1,019) = 7.36, *p* < 0.001, Cohen’s *f*^2^ = 0.05, power = 1.00. Vicarious online discrimination was negatively associated with internalized computing stereotypes, *b* = -0.10, *B* = -0.19, *t*(1,019) = -5.75, *p* < 0.001. Further, there emerged a significant interaction between direct online discrimination and vicarious online discrimination, *b* = -0.08, *B* = -0.18, *t*(1,019) = -3.69, *p* < 0.001. Specifically, for African American male and female adolescents who had low (-1 SD) or average (mean) frequency of vicarious online discrimination, the more frequently they direct online discrimination, the lower internalized computing stereotypes against the Black community they endorsed (*p*s < 0.01); however, this association was nonsignificant for those who had high (+1 SD) frequency of vicarious online discrimination. The three-way interaction among direct online discrimination and vicarious online discrimination and adolescent gender was nonsignificant. Contrary to Hypothesis 2a, these findings suggest that vicarious online discrimination to discrimination mitigated, rather than magnifying the internationalization of direct online discrimination, the moderating effects of which did not differ by adolescent gender as explored in Research Question 2a.

#### Moderating effects of parental technological attitudes

It was hypothesized that parental technological attitudes would mitigate the association between direct online discrimination and internalized computing stereotypes for adolescents (Hypothesis 2b); further, whether the moderating effect of parental technological attitudes would vary between male and female adolescents were explored (Research Question 2b). When parental technological attitudes were entered as one of the moderators, the regression model explained 4% of variance in internalized computing stereotypes, *R*^2^ = 0.04, *F*(7, 1,020) = 6.33, *p* < 0.001, although the effect size was small, Cohen’s *f*^2^ = 0.04, power = 1.00. Parental technological attitudes were negatively associated with their adolescent child’s internalized computing stereotypes, *b* = -0.13, *B* = -0.15, *t*(1,020) = -5.30, *p* < 0.001. Further, parental technological attitudes and adolescent gender moderated the association between adolescents’ direct online discrimination and internalized computing stereotypes, a three-way interaction, *b* = 0.08, *B* = 0.07, *t*(1,020) = 2.64, *p* = 0.009. Direct online discrimination was positively associated with internalized computing stereotypes for African American female adolescents whose parents had low (-1 SD) and average (mean) positive technological attitudes; however, this association was nonsignificant for those whose parents had high (+1 SD) positive technological attitudes. Parental technological attitudes did not affect the direct online discrimination – internalized computing stereotypes relation for male adolescents. Therefore, Hypothesis 2b was partially supported as we observed mitigating effects of parental technological attitudes for female but not male adolescents, highlighting gender differences in the moderating effects of parental technological attitudes (Research Question 2b).

#### Moderating effects of racial identity centrality

We also predicted that racial identity centrality would weaken the association between direct online discrimination and internalized computing stereotypes (Hypothesis 2c) and explored if adolescent gender would affect the interplay between racial identity centrality and direct online discrimination (Research Question 2c). The regression model with adolescents’ racial identity centrality entered as one of the moderators accounted for 25% of variance in internalized computing stereotypes, *R*^2^ = 0.25, *F*(7, 1,019) = 49.48, *p* < 0.001, and the effect size was medium, Cohen’s *f*^2^ = 0.37, power = 1.00. Adolescents’ racial identity centrality was negatively associated with internalized computing stereotypes, *b* = -0.34, *B* = -0.42, *t*(1,019) = -17.04, *p* < 0.001. Further, the three-way interaction among direct online discrimination, racial identity centrality, and adolescent gender was significant, *b* = 0.11, *B* = 0.12, *t*(1,020) = 5.02, *p* < 0.001. The direct online discrimination-internalized computing stereotypes association was positive only for African American female adolescents who reported low (-1 SD) levels of racial identity centrality, and this association was nonsignificant for female adolescents who reported average (mean) or high (+1 SD) levels of racial identity centrality, whereas racial identity centrality did not moderate this association for male adolescents. Another way to decompose the moderating findings was that racial identity centrality was negatively associated with internalized computing stereotypes, which held true for male adolescents regardless of their direct online discrimination whereas this association affected female adolescents who perceived high (+1 SD) frequency of direct online discrimination in comparison to those who perceived average (mean) or low (+1 SD) frequency of direct online discrimination. In summary, findings partially supported Hypothesis 2c such that racial identity centrality mitigated the internalization of direct online discrimination with a gendered effect (Research Question 2c), which was only significant for female adolescents.

## Discussion

Perceived direct discrimination based on race and gender can bring significant costs to the development and well-being of African American adolescents of color (Stevens-Watkins et al., 2014). Understanding their experiences in a rapidly evolving and engaging online environment offers insights into whether and how adolescents may be negatively affected by their perceptions of online discrimination. More importantly, adopting an ecological framework (Bronfenbrenner and Morris, 1998) via an intersectional lens (Crenshaw, 1991; McCall, 2001; Cho et al., 2013; Collins, 2015; Collins and Bilge, 2016), we examined how contextual and individual influences affect how adolescents may internalize direct online discrimination to negative beliefs about Black people and Black women’s computing abilities.

### Direct online discrimination and internalized computing stereotypes

The first goal of this study was to examine if adolescents’ direct online discrimination would be associated with greater internalized computing stereotypes (Hypothesis 1), and if this association would differ by adolescent gender (Research Question 1). Broadly, our findings revealed that those African American male and female adolescents who perceived more frequent online discrimination against their own identities endorsed greater stereotypes against the computing abilities in the Black community. This impact did not differ between male and female adolescents. This finding was consistent with Hypothesis 1 yet offered a slightly different pattern when compared to Tynes and colleagues’ previous findings (2012). Tynes et al. (2012) found that only African American female adolescents, but not male adolescents, reported lower general self-esteem as they perceived online racial discrimination. The difference between our findings and Tynes et al.’s might reflect the nuances in the measures we selected [i.e., discrimination based on race and gender in our study vs. racism in Tynes et al. (2012); internalized computing stereotypes as the outcome in our study vs. general self-esteem in Tynes et al. (2012)] and the representativeness of our samples [i.e., a national sample of over 1,000 adolescents in our study vs. 125 adolescents in Tynes et al. (2012)]. It also suggests that both African American male and female adolescents may be affected by direct online discrimination regarding frequency of occurrence and effects on internalized computing stereotypes.

As an intersectional approach reconceptualizes the meaning of individual and social identities, the research focus is shifted toward identifying and understanding the mechanisms by which inequalities are created and expressed within those categories (Else-Quest and Hyde, 2016). It is thus important to recognize that direct online discrimination based on race and gender as measured in study (i.e., same language) may have different underlying meanings and interpretations by male and female adolescents. To further illustrate and examine how the impact of direct online discrimination may be contextualized, we focused the second goal of this study on examining systemic and individual factors that may affect and moderate the internalization of direct online discrimination from an ecological lens (Bronfenbrenner and Morris, 1998).

### Moderating effects of vicarious online discrimination

There emerged partial support for all proposed factors that influence the internalization of direct online discrimination for African American adolescents. Specially, at the exosystem level, we investigated the effects of vicarious online discrimination (Hypothesis 2a) by adolescent gender (Research Question 2a). Contrary to prior literature adopting an experimental design on exposure to sexism (Eliezer et al., 2010), we found that greater vicarious online discrimination mitigated the likelihood for African American male and female adolescents to adopt stronger internalized computing stereotypes in the face of more frequent direct online discrimination. Given that over 71% of adolescents reported experiencing or witnessing online discrimination (Tynes et al., 2008), adolescents appeared to report more frequent vicarious online discrimination than direct online discrimination against their personal identities. One possibility is that having vicarious online discrimination may imply that adolescents are engaging in online spaces more frequently because they feel confident with their computing abilities (i.e., weaker internalized computing stereotypes). They may also encounter more general discrimination against the Black community and have habituated to such occurrences because they have chosen to engage in more spaces that target Black audiences (Dixon et al., 2009). Further, drawing from a priming paradigm (Appiah, 2004), adolescents who are exposed to more frequent general online discrimination may have greater opportunities to deepen their understanding of what and how discrimination in an online context occurs. They may develop effective coping strategies from vicarious online discrimination, and therefore, foster a greater agency to detect discrimination against themselves and cope with it more effectively. In other words, having a greater awareness of online discrimination due to vicarious online discrimination may have promoted adaptive coping strategies (Hope et al., 2015), especially as adolescents engage with more similarly-minded individuals online (Keum and Miller, 2018).

### Moderating effects of parental technological attitudes

At the microsystem level, parental technological attitudes were predicted to weaken the positive association between direct online discrimination and internalized computing stereotypes (Hypothesis 2b); however, this effect was only significant for African American female but not male adolescents (Research Question 2b). On one hand, the lack of significant findings on the moderating effects of parental technological attitudes for male adolescents could be associated with the gendered parenting practices documented in prior literature (Varner and Mandara, 2013; Skinner et al., 2016). African American male adolescents tend to be encouraged to explore their autonomy across different realms of life (e.g., Varner and Mandara, 2013; Skinner et al., 2016). Therefore, they may be less positively influenced by their parents’ affirming attitudes specifically toward technology.

On the other hand, African American female adolescents are more likely to be monitored especially when there is potential threat of discrimination in the online world, so their parents’ optimistic outlook on technology may have shed a more salient positive light. Parents who endorse positive technological attitudes may be inclined to engage in more cohesive communication with their adolescent children (Kim and Davis, 2017) and convey racial and ethical socialization messages that are related to greater self-esteem in adolescents (e.g., Murry et al., 2009). They may also provide messages to African American female adolescents about what it means to be both African American and female (Thomas and King, 2007) and how to cope with online spaces that may not value either social identity. Together, findings highlight the importance of considering and examining interpersonal relationships as they affect adolescents’ access to and knowledge about power and oppression within online interactions (Collins, 2000).

Notably, internalized computing stereotypes may prevent African American female adolescents from entering or persisting in a technology-related field. Therefore, positive parental technological attitudes may not only facilitate African American female adolescents to resist negative stereotypes against Black people and Black women in technology, but also minimize potential barriers for female adolescents to engage in technology or related fields in the future.

### Moderating effects of racial identity centrality

At the individual level, we examined if adolescents’ racial identity centrality would weaken the direct online discrimination-internalized computing stereotypes association (Hypothesis 2c) by adolescent gender (Research Question 2c). In partial support of Hypothesis 2c, African American female, but not male adolescents’ racial identity centrality mitigated the internalization of direct online discrimination. The more central and important African American female adolescents consider their racial identity as to their self-image, the more certain they may be about themselves, the more likely they may visit online spaces that gear toward people of their identities (Dixon et al., 2009), and the more equipped they may have become to cope with negative online encounters. This adheres to a resilience framework such that racial identity centrality may serve as an asset to assist female adolescents to internally process direct experiences with online discrimination. Racial identity centrality may also instill agency for female adolescents to change the online landscape, so they may be more likely to perceive and resist oppression as online discrimination become expected but can be effectively dealt with. So they are less likely to internalize negative messages against technological abilities solely based on their identities. In addition, an online context can provide adolescents fast and far-reaching resources and opportunities to enact change and actions (e.g., learning from and connecting with other African American women and female adolescents, and following and joining events pertaining to liberation).

The findings for African American male adolescents suggest that racial identity centrality may not affect the association between direct online discrimination and internalized computing stereotypes similarly as that for female adolescents. However, male adolescents who endorse greater racial identity centrality appear to endorse lesser negative beliefs about the Black communities, regardless of the frequency of direct online discrimination. In other words, racial identity centrality has a direct impact, rather than a buffering effect, on male adolescents’ internalized computing stereotypes. One interpretation for this gender difference may be that for male adolescents who report greater racial identity centrality, they may have become accustomed to and adopted effective coping for discrimination, which assisted them in being less affected by its negative impact on internalized computing stereotypes (Brondolo et al., 2009; Szymanski and Lewis, 2016). This is different from previous findings on how male adolescents who endorse stronger racial identity centrality may be more susceptible to the negative messages about themselves (Brondolo et al., 2009; Szymanski and Lewis, 2016). Alternatively, Brondolo et al. (2018) proposed a biopsychosocial framework of understanding how biological, psychological, and social factors may affect individuals’ perceived discrimination (offline and online) and health. Given this study focuses on psychological and social factors, it is also possible that the observed gender differences may be affected by underlying physiological differences in stress responses between male and female adolescents.

Together, findings in this study suggest that oppression does not automatically mean disempowerment. Rather, the role of racial identity centrality may be more nuanced as African American adolescents are continuously exploring their racial and gender identities (Umaña-Taylor et al., 2014). Future research may explore additional explanations of our findings by studying the underlying mechanism that affected adolescents use of the Internet and digital media as related to their racial identity (e.g., if those viewing racial identity as more central choose to engage in online spaces in different ways compared to those viewing racial identity as less central or important).

## Strengths and limitations

This study provides important and promising implications in response to a recent call to enhance our understanding of African American adolescents’ experiences with discrimination in the digital world (Keum and Miller, 2018). With a large national, geographically diverse sample of African American adolescents and their parents, our findings reflect an examination of the experiences of African American families of diverse background in terms of socioeconomic status, geographic locations, and familial composition (i.e., single versus dual-parent households). Adopting an ecological framework via an intersectional lens, we have afforded the opportunity to embed the internalization of direct online discrimination within multi-faceted systems and explore similarities and differences between African American male and female adolescents.

Several limitations are noteworthy in interpreting findings. First, we only assessed frequency of direct online discrimination and vicarious online discrimination. We did not assess adolescents’ perceived level of stress from these experiences. The effects of frequency and stress of direct online discrimination offline may vary. Additional measurement concerns include the use of a single item to assess racial identity centrality and the use of two items to assess direct online discrimination as they may bring limitations to measurement reliability and validity, which should be recognized in interpreting findings. Researchers may consider validating potential efficacy for the use of a single item on racial identity centrality, similar to the validated use of a single item on health and quality of life (Cunny and Perri, 1991; Hyland and Sodergren, 1996). Data skewness on internalized computing stereotypes and racial identity centralization should also be considered in interpreting the results.

Second, our operationalization of intersectionality as a conceptual and methodological framework does not fully represent the unique position African American male and female adolescents are placed in the online world. Both qualitative (Gray, 2012) and quantitative research (Szymanski and Lewis, 2016) has suggested that African American male and female adolescents may experience gendered racism, raced sexism, or discrimination based on the intersections of a myriad of identities (e.g., race, gender, socioeconomic status, sexual orientation geographic locations, religious beliefs). Our findings with a focus on direct online discrimination assessed by perceived disrespect and mistreatment based on race and gender are in response to the methodological challenges in quantitative intersectionality research (Cole, 2009) and serve as a proximal estimate. It should also be acknowledged that online discrimination was measured in relatively broad terms in this study. Our findings cannot be applied to understanding discrimination based on the other identities adolescents endorse.

Third, we focus on parental technological attitudes as a part of the influences in the microsystem, and more specifically on how positively parents consider technology. Although we attempt to refer our findings to prior research predominantly on parenting behaviors and ethnic and cultural socializations, caution is needed in the interpretations. We do not, for instance, examine the relationship dynamics between father-sons and father-daughters. This limitation is due, in part, to the underrepresentation of such dyads in our sample. As such, we conducted analyses based on adolescent gender instead of parent-child dyadic composition. Consequently, we do not know how a more balanced sample involving more fathers would have changed the overall study results. Additional caution is needed given the overall effect size of our findings tended to be small, with the exception for the medium effect size of the finding on the moderating effect of adolescents’ racial identity centrality. It may be helpful to note that domains of the measures used in this study were developed based on most common themes discussed by parents and adolescents in focus groups during the first phase of the larger project. The description of being “good at using computers” assessed on the internalized computing stereotypes scale can be interpreted in multiple ways, in terms of being good at social media use, IT services, and computer repair, etc. More specific items relative to all the ways one can be “good at using computers” may be helpful in future studies. The small effect sizes may be influenced by the broad nature of assessment on direct and various online discrimination, and the unique focus of internalized computing stereotypes on negative views of technological abilities for African Americans in general compared to those of other races and for African American women/girls compared to men/boys. There may also be influences from generally low scores on internalized computing stereotypes, and future studies may benefit from including adolescents who endorse a greater range of internalized computing stereotypes.

Finally, the Ecological Systems Model (Bronfenbrenner and Morris, 1998) posits that the multiple systems adolescents live in interact with each other. We chose to focus on the interplay between each systemic and individual factors (i.e., exosystem by individual factors, microsystem by individual factors). We want to acknowledge the complex interplays contextual factors may have with one another (e.g., excosystem by microsystem factors) and among more than two layered systems (e.g., excosystem by microsystem by individual factors), which are, however, beyond the scope of our current analyses.

## Implications and future directions

Findings in this study highlight that both African American male and female adolescents are likely to internalize online discrimination about their racial and gender identities with more frequent direct online discrimination. Although adolescents may gain increased confidence in their abilities and interests in technology with increased use of digital and social media, their perceptions of online discrimination against their personal identities may place them at risk for more negative views of their technological selves. Using an intersectional analysis allows us to recognize standpoints of African American adolescents. African American women’s knowledge and voices have historically been dismissed and undermined given their standpoints of being African American (not White) and women (not men), from a perspective of Afrocentric feminist epistemology or Black feminist epistemology (Collins, 2000). This lens aligns with an intersectionality framework to highlight that positions within intertwined oppressed socioeconomic groups (e.g., in regard to race, gender, socioeconomic status) affects individuals’ access to power and privilege and how their knowledge and voices are consequently acknowledged. Therefore, it is important to attend to African American women’s and female adolescents’ voices and experiences while considering the historical contexts of oppression. To this end, some scholars have posited that acknowledging the oppressed position of Black women against dominant groups as beneficial (e.g., Sprague, 2001) whereas others argued for the risks associated with ranking of marginalized groups (e.g., Collins, 2000). Nonetheless, findings in this study highlight that it is critical to understand how the unique sociocultural background of African American male and female adolescents interact with their experiences with digital media. Future research is urged to adopt a standpoint of alliance and coalitions among oppressed groups to examine adolescences’ development in the digital world in addition to the more well-studied, offline setting (Keum and Miller, 2018).

We also observed multiple resilience factors for adolescents in the face of online discrimination. The mitigating effect of vicarious online discrimination for both male and female adolescents warrants further research. More systemic attention to the influences of the digital environment is needed to promote healthier development for adolescents, especially African American adolescents. More research like this can help inform a greater audience of the negative impact of personal experiences with online discrimination to advocate for potential policy changes to promote adaptive coping for adolescents facing such encounters.

Prospective research may build upon our findings to assess other aspects of online experiences as well as the interplay between online and offline experiences. For example, in addition to online mistreatment, are African American adolescents aware of any individual or collective actions that aim to change the oppressive digital culture? Have they been provided opportunities to use online platforms to engage in resistance of and liberation from oppression, which may translate to offline actions? Perceived offline discrimination has been found to poise African American and Latinx adolescents to perceive more frequent online discrimination, but not vice versa (Tynes et al., 2020). How do adolescents traverse different online and offline spaces with different social structures and power dynamics (Keum and Miller, 2018)?

Future research can also examine if multifactorial influences assessed in this study are related to adolescents’ agency to change and intent to persist in the technological field despite prevalence of negative stereotypes. From an intersectional lens, how direct online discrimination may affect adolescents’ narratives about their experiences and their agency to change is equally important as how adolescents’ narratives affect their perceptions of discrimination and positive messages. In the context of media coverage of policy brutality, although adolescents may be exposed to discriminatory content reflecting ignorance, they may also view positive and possibly inspiring content on the Black Lives Matter movement online and connect with communities of individuals with similar beliefs and experiences (Keum and Miller, 2018; Rogers et al., 2021). Researchers are encouraged to examine the nuances in online exposure for African American adolescents, as they may experience mixed messages in support of and against their identities and beliefs. Further, future studies are urged to adopt an intersectional lens to assess the associations between media and adolescents’ agency to change or persist.

Valkenburg et al. (2016) have highlighted how the new media environment driven by the expansion of the Internet and individuals’ use of the mechanism to interact has led to different types of media effect theories and research. One example is computer mediated communication (CMC) theories, which focus on “questions such as whether, and how, certain characteristics of CMC, such as anonymity or the lack of nonverbal (auditory or visual) cues, influence the quality of social interaction and the impressions CMC partners form of one another” (Valkenburg et al., 2016, p. 328). Less known and studied in CMC is transactional media effects theories which examine how sending media content affects both the receiver and the sender. What we do not know is how African American adolescents’ interactions with negative content such as online discrimination based on race and gender affect their interpersonal and intrapersonal processes. With the overabundance of media stories about George Floyd’s public execution amongst many other oppressive stories African Americans encounter on a daily basis, the time is ripe to conduct such research. We believe the intrapersonal is incredibly important given that the sender’s civic messaging influenced their civic engagement (Shah et al., 2005). What is comforting, given the tenor of the times, is that the self-generated messages had a greater effect than a sender observing traditional media. What this suggests is the images of Black men and women being victims of institutional racism may not disempower adolescents as much as believed. Instead, if provided opportunities to identify and perform their agency in CMC, adolescents may indeed become digital protestors in their self-perception and influence others through online interpersonal relations. These perspectives can also inform parents’ technological attitudes to encourage adolescents to maximize online spaces for the development of their self-identity and desire to become change agents in social and technological domains.

Additionally, findings in this study may inform educational or intervention programs that take a systemic and/or culturally responsive framework to promote positive attitudes for African American female adolescents’ parents and the exploration of racial identity for female adolescents themselves. Researchers can shed light on the quality of parent-adolescent communication in addition to parental attitudes (Cooper and McLoyd, 2011) and other aspects of racial identity in addition to centrality (e.g., public or private regard; Sanchez and Garcia, 2009) as potential protective factors for direct online discrimination and assess the unique roles mothers and fathers play in dual- and single-parent households. Importantly, the associations between direct online discrimination, systemic and individual factors (e.g., vicarious online discrimination, parental technological attitudes, adolescents’ racial identity centrality), and internalized computing stereotypes are not linear. These factors may affect each other across time, which warrants longitudinal designs and qualitative inquires of adolescents’ direct and vicarious experiences with media coverage of critical events.

## Conclusion

Our findings highlight that African American male and female adolescents’ experiences online are unique and influenced by multifactorial systems. Overall, both African American male and female adolescents who perceive more frequent direct online discrimination against their race and gender tend to endorse stronger computing stereotypes or negative beliefs about African Americans’ abilities to engage in technology or related jobs. On the other hand, adolescents may be less vulnerable to these negative impacts as they are more frequently exposed to vicarious online discrimination, whereas African American female adolescents are specifically, less likely to be negatively affected by direct online discrimination if their parents uphold positive attitudes toward technology and if they themselves consider their racial identity central to their self-image. It is likely that having a warm and supportive familial environment by parent(s) who promote exploration of adolescents’ own racial identity and digital media use may have cascading benefits for their identity and technological development. African American adolescents, though historically marginalized in the digital culture, are becoming more active users of digital and social media (e.g., Rideout et al., 2011; Tynes and Mitchell, 2014; Nielsen, 2016). We urge prospective research to replicate and build upon our findings to deepen our understanding of African American adolescents’ intragroup experiences as well as systemic and individual factors that promote positive interactions with digital media.

## Data availability statement

The raw data supporting the conclusions of this article will be made available by the authors, without undue reservation.

## Ethics statement

The studies involving human participants were reviewed and approved by Arizona State University’s Institutional Review Board (IRB). The patients/participants provided their written or verbal informed consent to participate in this study.

## Author contributions

CT formulated the research questions for this manuscript, conducted data analyses, and drafted the manuscript. KS conceptualized the current project, led the research team for this project which collected the data, and provided conceptual feedback, and edits on the manuscript. Both authors contributed to the article and approved the submitted version.
